# Severe Hypocalcemia Occurring During the Hospitalization of a Patient Affected by Permanent Post-Surgical Hypoparathyroidism with Multimorbidity: A Case Report

**DOI:** 10.2174/0118715303324351240725071502

**Published:** 2024-08-06

**Authors:** Isabella Nardone, Sium Wolde Sellasie, Alessandra Cinque, Giovanni Tacchi, Simona Zaccaria, Cristina Giusto, Roberto Palumbo, Achille Gaspardone, Luigi Uccioli, Stefano Amendola

**Affiliations:** 1 Division of Endocrinology and Diabetes, CTO Andrea Alesini Hospital, Department of Biomedicine and Prevention, University Tor Vergata, Rome, 00133, Italy;; 2 PhD School of Applied Medical-Surgical Sciences, University of Rome Tor Vergata, Rome, 00133, Italy;; 3 Division of Cardiology, Sant'Eugenio Hospital, Rome, 00144, Italy;; 4 Thyroid Endocrine Surgery, Sant’Eugenio Hospital, Rome, 00144, Italy;; 5 Thoracic Surgery Unit, Campus Bio-Medico University, Rome, 00128, Italy;; 6 Unit of Nephrology, Sant'Eugenio Hospital, 00144, Rome, Italy

**Keywords:** Hypoparathyroidism, hypocalcemia, QT interval prolongation, calcitriol, chronic replacement therapy, thyroidectomy

## Abstract

**Background:**

Patients with permanent hypoparathyroidism experience an impaired quality of life, due to acute and chronic complications that may affect several organs, with an increased risk of hospitalisation and death. Adequate and continuous replacement therapy with calcium and calcitriol is necessary to avoid symptoms and long-term complications related to hypocalcemia.

**Case Presentation:**

A 63 years old male, affected by permanent post-surgical hypoparathyroidism, was hospitalized in the cardiology department because of a dehiscence of the subcutaneous housing of the double-chambered implantable cardioverter-defibrillator. Chronic replacement therapy for hypoparathyroidism was poorly controlled and, during hospitalization, severe hypocalcemia occurred together with electrocardiographic and echocardiogram life-threatening alterations.

**Conclusion:**

Constant and targeted long-term replacement therapy with calcium and particularly calcitriol is necessary to avoid major consequences on patients’ health, especially during acute events and in the presence of other comorbidities.

## 1 INTRODUCTION

Post-thyroidectomy long-term hypoparathyroidism occurs in 5-16% of cases, with principal risk factors being concomitant level VI lymph node dissection, reoperative surgery, and inter-surgeon variation [1-3]. The incidence of permanent post-surgical hypoparathyroidism in Italy is estimated to be 27/10.000 inhabitants, even if the real burden of the disease may be underestimated [4]. Patients with permanent hypoparathyroidism experience poor quality of life due to acute and chronic complications affecting several organs, with an increased risk of hospitalization and death [5]. Patients with poorly controlled hypoparathyroidism need access to the emergency department and need hospitalization more frequently, with strong repercussions also on healthcare costs [6]. The main acute complication in this setting is represented by acute hypocalcemia. The latter is associated with reversible cardiac dysfunction [7, 8], supported by QT interval prolongation, sinus tachycardia, and heart failure [7, 8]. For this reason, adequate replacement therapy with calcium and calcitriol is essential to avoid symptoms and long-term complications related to hypocalcemia [9-11]. In particular, calcitriol, whose synthesis depends physiologically on the levels of parathyroid hormone (PTH), is essential for the absorption of calcium and phosphate from the gastrointestinal system and for maintaining calcium levels within the normal range [12], especially during illness states and in the presence of other comorbidities.

## 2 CASE PRESENTATION

A 63-years-old male, affected by chronic ischemic heart disease and with a subcutaneous double-chambered implantable cardioverter-defibrillator (ICD), implanted for dilated heart disease, was hospitalized in the Department of Cardiology due to dehiscence of the subcutaneous housing of the device. The patient was also affected by diabetes mellitus, chronic kidney disease, and arterial hypertension. In 1993, he had undergone a total thyroidectomy due to Basedow-Graves disease, complicated by permanent post-surgical hypoparathyroidism; at the moment of the hospitalization, he was taking levothyroxine 100 mcg and calcium carbonate 1000 mg twice daily, but the latter one with scarce adherence and without endocrinological follow-up. His chronic therapy was completed by furosemide 25 mg twice a day, acenocoumarol, lansoprazole 30 mg, bisoprolol 2,5 mg, irbesartan 150 mg, atorvastatin 40 mg, ezetimibe 10 mg, canrenone 50 mg, and insulin with basal-bolus scheme. The patient followed a varied diet without specific restrictions. The assessment on arrival did not reveal acute symptoms. Physical examination showed a purulent discharge from the surgical wound; blood pressure was 135/90 mmHg, with a heart rate of 98 beats per minute. Neurological examination was unremarkable. Bedside echocardiography revealed a 45% ejection fraction, with no evidence of endocardial formations. Laboratory investigations (Table **1**) showed an increase in inflammation indices and white blood cells with a prevalence of neutrophils. Calcium blood levels were 6.8 mg/dl, which is a testimony to the suboptimal adherence to replacement therapy for hypoparathyroidism. In fact, blood tests relating to the years preceding hospitalization showed calcium values chronically around 6-7 mg/dl. Acute calcium replacement therapy was started by intravenous infusion.

**Fig. (1):**
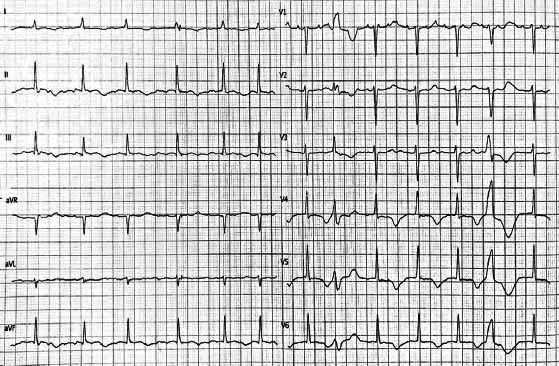
Electrocardiogram (ECG) showing QT interval prolongation.

**Fig. (2):**
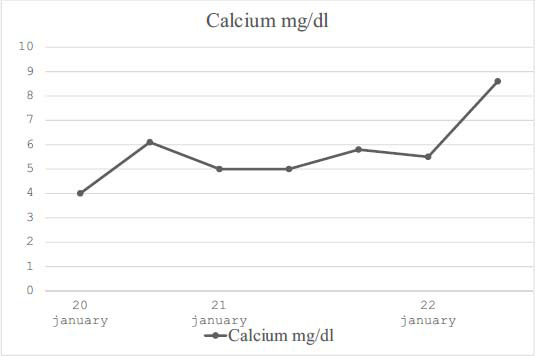
Trend of calcium values during the hospitalization; # start of calcitriol therapy.

Subsequently, a surgical review of the implantation site of the device was carried out. The microbiological analysis of the wound swab was positive for *Staphilococcus anginosis*; specific antibiotic therapy with Ceftriaxone was established. The day after, the patient manifested confusion, muscle pain, and tingling. The electrocardiogram (ECG) showed a prolonged QT interval of 640 milliseconds (≤430-450) (Fig. **1**). Bedside echocardiography revealed a decrease in ejection fraction (35%). Laboratory tests were carried out, showing a condition of severe hypocalcemia, with a calcium concentration of 4.0 mg/dl (calcium corrected with albumin 4.8 mg/dl). Further investigation found serum intact parathyroid hormone of 11.9 pg/ml (reference range 15.0 - 83.1 pg/ml). Intravenous calcium replacement therapy was enhanced but without significant benefit. In fact, the laboratory tests still showed levels of calcium of 6.1 mg/dl. Despite the optimization of calcium replacement, adequate blood concentrations could not be reached. Endocrinological consultation was requested to re-assess postoperative hypoparathyroidism: the patient was taking only calcium, without calcitriol; therefore, calcitriol, 0.5 mcg twice daily was started. With careful interval ECG and laboratory monitoring, calcium concentrations normalized (8.6 mg/dL) in the following two days, and symptoms and ECG changes were resolved (Fig. **2**). Calcium infusion was stopped; oral calcium carbonate and calcitriol were continued and confirmed in discharge therapy.

## 3 DISCUSSION

This case report highlights how permanent hypoparathyroidism represents a clinical issue, which, if not well treated, can lead to important complications and serious repercussions on patients' health. Over the years following the surgery, the patient was hospitalized several times due to heart disease, and his chronic therapy was often modified. The replacement treatment for parathyroid function was not optimized for a long time. Furthermore, he did not have good compliance with calcium supplementation. In fact, blood tests before hospitalization showed calcium values around 6-7 mg/dl on several occasions. Chronic inadequate blood calcium levels may have contributed, together with diabetes mellitus and other comorbidities, to the progression of renal failure and the development of cardiovascular diseases [8, 9, 13]. The impaired kidney function may have further compromised the production of active vitamin D [10]. During hospitalization, probably due to the stress related to the infection, poor food intake, and the lack of calcitriol replacement, the calcium values reached extremely low levels. The patient's symptoms were not particularly serious; in fact, he has had chronically low calcium values for many years; however, worsening of heart failure occurred with a decline in ejection fraction and important electrocardiographic signs. Calcemic levels began to rise significantly only after the start of oral calcitriol therapy, with a resolution of the electrocardiographic alterations and normalization of ejection fraction. This case report confirms the importance of setting a correct and personalized replacement therapy, establishing a systematic follow up, to prevent and research the chronic complications of this condition [8, 9, 14]. Unfortunately, the follow-up of chronic hypoparathyroidism revealed challenges, especially many years after surgery and in the presence of other significant health problems. In light of the serious acute and chronic consequences that it can cause, it is necessary to increase the levels of attention towards the disease and its correct management. Particular emphasis should be placed on making the patients aware of the importance of replacement therapy and investigating, at the same time, their adherence to the prescription. Guidelines updated in 2022 [9] suggest treating with calcium citrate or carbonate (the dose varies between 500-3000 mg divided in two or three doses, preferably with meals) and an active vitamin D analogue (0.25-3 μg per day, divided into several doses), in order to bring calcium values back to the lower part of the normal range.

## CONCLUSION

Hypocalcemia due to post-surgical hypoparathyroidism can have serious repercussions on the general health of the individual, increasing the risk of incurring both acute and chronic complications, hospitalization, and death. This case report stresses the importance of paying particular attention, during hospitalization and acute events, to patients suffering from permanent hypoparathyroidism, particularly in those with several comorbidities, as hypocalcemia can significantly contribute to the worsening of clinical conditions.

## AUTHORS’ CONTRIBUTIONS

The authors confirm their contribution to the paper as follows: study conception and design: R.P., A.G., L.U., S.A.; data collection: A.C.; data analysis and interpretation: S.W.S., S.Z., C.S.; writing the paper: I.N., G.T. All authors reviewed the results and approved the final version of the manuscript.

## Figures and Tables

**Table 1:** Laboratory test at the admission.

Laboratory Test	Blood Values	Reference Range
Haemoglobin	9.9 g/dl	14 - 18
White blood cells	10.40 x 10^3^/µL	4 - 10
Neutrophils	7.19 x 10^3^/µL	1.80 – 7.0
Urea	82 mg/dl	18 - 55
Creatinine	1.72 mg/dl	0.73 – 1.18
Glomerular filtration rate	41 ml/min	≥ 90
Glucose	143 mg/dl	70 – 110
C-reactve protein	13.38 mg/dl	0.00 – 0.50
TSH	0.67 µUI/ml	0.35 – 4.94
fT4	1.13 ng/dl	0.70 – 1.48
AST	24 U/L	5 - 34
ALT	11 U/L	0 – 55
Albumin	30 g/L	32 - 46
Calcium	6.8 mg/dl	8.4 – 10.2
Magnesium	1.7 mg/dl	1.6 – 2.6
Phosphorus	4.3 mg/dl	2.3 - 4.7

**Abbreviations:** Thyroid-stimulating hormone (TSH), free thyroxine (fT4), aspartate aminotransferase (AST), and alanine aminotransferase (ALT).

## Data Availability

The data that support the findings of this study are available from the corresponding author upon reasonable request.

## LIST OF ABBREVIATIONS

ALT: Alanine Aminotransferase
AST: Aspartate Aminotransferase
ECG: Electrocardiogram
fT4: Free Thyroxine
ICD: Implantable Cardioverter-Defibrillator
PTH: Parathyroid Hormone
TSH: Thyroid-Stimulating Hormone

## STANDARDS OF REPORTING

CARE guideline were followed in this study.
